# Evaluating the accuracy of amplicon-based microbiome computational pipelines on simulated human gut microbial communities

**DOI:** 10.1186/s12859-017-1690-0

**Published:** 2017-05-30

**Authors:** Jonathan L. Golob, Elisa Margolis, Noah G. Hoffman, David N. Fredricks

**Affiliations:** 1 Vaccine and Infectious Disease Division, Fred Hutch, 1100 Eastlake Ave E, E4-100, Seattle, WA 98109 USA; 2 Seattle Childrens Hospital, Seattle, WA USA; 3 Department Laboratory Medicine, University of Washington, Seattle, WA USA; 4 Division of Allergy and Infectious Diseases, University of Washington, Seattle, WA USA

**Keywords:** Microbiome, Classification, Operational taxonomic unit, Optimization, UniFrac, QIIME, MOTHUR

## Abstract

**Background:**

Microbiome studies commonly use 16S rRNA gene amplicon sequencing to characterize microbial communities. Errors introduced at multiple steps in this process can affect the interpretation of the data. Here we evaluate the accuracy of operational taxonomic unit (OTU) generation, taxonomic classification, alpha- and beta-diversity measures for different settings in QIIME, MOTHUR and a pplacer-based classification pipeline, using a novel software package: DECARD.

**Results:**

In-silico we generated 100 synthetic bacterial communities approximating human stool microbiomes to be used as a gold-standard for evaluating the colligative performance of microbiome analysis software. Our synthetic data closely matched the composition and complexity of actual healthy human stool microbiomes. Genus-level taxonomic classification was correctly done for only 50.4–74.8% of the source organisms. Miscall rates varied from 11.9 to 23.5%. Species-level classification was less successful, (6.9–18.9% correct); miscall rates were comparable to those of genus-level targets (12.5–26.2%). The degree of miscall varied by clade of organism, pipeline and specific settings used. OTU generation accuracy varied by strategy (closed, de novo or subsampling), reference database, algorithm and software implementation. Shannon diversity estimation accuracy correlated generally with OTU-generation accuracy. Beta-diversity estimates with Double Principle Coordinate Analysis (DPCoA) were more robust against errors introduced in processing than Weighted UniFrac. The settings suggested in the tutorials were among the worst performing in all outcomes tested.

**Conclusions:**

Even when using the same classification pipeline, the specific OTU-generation strategy, reference database and downstream analysis methods selection can have a dramatic effect on the accuracy of taxonomic classification, and alpha- and beta-diversity estimation. Even minor changes in settings adversely affected the accuracy of the results, bringing them far from the best-observed result. Thus, specific details of how a pipeline is used (including OTU generation strategy, reference sets, clustering algorithm and specific software implementation) should be specified in the methods section of all microbiome studies. Researchers should evaluate their chosen pipeline and settings to confirm it can adequately answer the research question rather than assuming the tutorial or standard-operating-procedure settings will be adequate or optimal.

**Electronic supplementary material:**

The online version of this article (doi:10.1186/s12859-017-1690-0) contains supplementary material, which is available to authorized users.

## Background

Complex microbial communities colonize and affect a variety of environments, including our own bodies. Next-generation sequencing of amplicons from a taxonomically informative gene (like the small subunit ribosomal RNA gene) is useful for estimating the composition of microbial communities and has been widely applied in diverse environments. Evaluating and optimizing the accuracy of this technique requires a gold standard for which one knows the true composition of the community.

Popular software packages for microbiome studies include QIIME [1] and MOTHUR [2]. The flow for most microbiome software is similar. The amplicon sequences are clustered into operational taxonomic units (OTUs)—sequences with sufficient similarity to be considered as arising from the same organism in the initial community. Analysis can proceed at that level, associating clinical outcomes with the presence or absence of a given OTU, calculating microbial alpha-diversity (richness and evenness) of the community, or beta-diversity (distance) between communities, with the OTU as a marker. Researchers often proceed to a classification step to identify each OTU as representing a given already-known organism in a shared reference database. This process can connect the OTU sequences to the larger body of microbiological research, converting associations into a deeper understanding of the members of the community and their capabilities. Even within a given analysis pipeline, there are a variety of settings to be selected: Which OTU generating strategy should be used; which clustering algorithm; which classifier and reference database?

Using constructed mock-communities as a gold-standard allows for a detailed assessment of the effects of DNA storage, extraction, PCR enzymes and primers, sequencing technique and classification software. The DNA extraction technique and PCR conditions dramatically affect accuracy of the technique more than sequencing platform, and in ways that are not easily addressed by software [3–5]. Community composition can affect the reliability of the results [6] and result in bias, with more complex communities particularly challenging [7]. Spiked in DNA into real samples has been successfully employed to test beta-diversity measuring techniques [8]. Standardized mock communities have been created to facilitate future work in this productive area [9].

In-silico data can serve as a gold standard as well, allowing uncultivated organisms and more complex communities to be considered, something not possible or practical with mock communities. Using in-silico simulations, early clustering algorithms were found to be overly stringent when generating OTUs [10]. The different alignments produced by references databases affected the quality of the downstream results [11]. Average neighbor clustering algorithms performed better in OTU generation [12], with large differences in output between algorithms [13]. The Clostridiales order was identified as particularly challenging for software to properly cluster [14]. In-silico data has been used to optimize the PCR primer selection process [15, 16] and identify misidentified sequences [17].

Despite all of this excellent work, it remains a challenge for a researcher performing a microbiome experiment, a reviewer critically evaluating a study for publication, or a reader considering the validity of the study to determine which pipeline, selected OTU strategy, reference database and classification tactics are the best—or even adequate in accuracy and precision—to support the conclusions of the study. In most papers, the standard methods described in the tutorials for the respective pipelines are used.

Here, we developed a software package DECARD (Detailed Evaluation Creation and Analysis of Read Data) to generate realistic synthetic datasets for which we have a known source of the sequences to be used as a gold standard when evaluating microbiome analysis software. We used DECARD to synthesize in-silico communities that approximate those we observe in healthy human stool to test the colligative performance of different microbiome analysis pipelines and settings in an idealized setting of no novel organisms and perfect PCR and sequencing or limited simulated sequencing and PCR errors. We performed in-silico PCR followed by simulated sequencing of the amplicons. The resultant amplicons were classified with QIIME, MOTHUR and a pplacer-based [18] classifier. We compared the outputs of each classification method against the true origins of the amplicons. We assessed for robustness, accuracy and resolution. All experiments were done with simulated MiSeq and 454-style amplicons, with and without simulated sequencing errors. Unless specified, results were similar for 454 and MiSeq, with or without simulated sequencing errors.

## Results

### Synthetic community generation

**Fig. 1:**
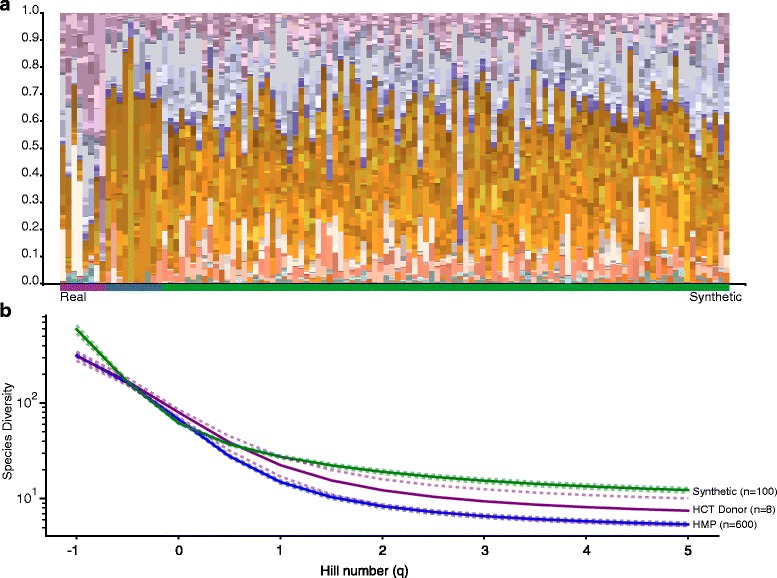
Estimated Real versus Synthetic Health Human Stool Microbiota. **a** Each column represents one sample. Each band represents one organism. The height of each band of color is proportional to the relative abundance of each sequence type. Taxonomically similar organisms are closer in color. Colors are by phylum (inspired by a gram stain): *Blue* and *purple* for *Firmucutes*; orange for *Bacteroides*; *Tan* and *pinks* for *Proteobacteria*. Estimated relative abundances from real data are on the left and *underlined* in *purple* for healthy donor human stool microbiota, *blue* for the human microbiome project samples; synthetic data is on the right, and *underlined* in *green*. **b** The diversity of the each microbiota (synthetic in *green*, healthy donor in *purple* and Human Microbiome Project (HMP) in *blue*) for Hill numbers varying from −1 to 5, in 0.5 intervals. *Solid lines* are the mean, and dashed lines span the 95% confidence interval after bootstrapping 5000 iterations (with replacement) for the mean

We generated 100 communities with a composition (specific clades of organisms, down to the genus level) and diversity (evenness and richness) similar to our estimates of normal stool. We used data from the healthy-gut cohort of the human microbiome project and our own samples from healthy donors to estimate the composition of a typical gut microbiota and define mathematical parameters (mean fractional abundance, standard deviation of fractional abundance, and number of species to be represented per genus) suitable for the DECARD “generate target module” (Additional file 1: Tables S1–S4). Figure 1a shows the community profile of the real stool microbiome data as compared to the synthetic communities, demonstrating similar representations of clades between our synthetic and real data. For diversity we used the approach suggested by [19], calculating diversity scores across Hill values of −1 to 5, with results shown in Fig. 1b. As we intended, at the extremes of the Hill value (low emphasizes rare organisms, high dominant organisms), our simulated populations had a higher diversity than the estimates from real data from healthy human stool (from the human microbiome project and stool samples from eight healthy donors). In the core range of Hill numbers from zero to one (the latter approximating the exponent of Shannon Diversity) our synthetic data closely matches that of the real data.

For each amplicon we know the true origin organism (represented by a full-length unambiguous 16S sequence from a reference organism deposited in the NCBI microbial 16S database on Silva database), with an associated full taxonomy.

### OTU generation

We then asked how well the various pipelines were at forming operational taxonomic units or OTUs. Each OTU (or clustered-together set of sequences) is meant to represent an organism in the initial community, suitable for unit measures of community diversity, for correlation analysis and for classification to a named organism.

There are three broad strategies used to generate OTUs: Closed OTU generation strategies align to a reference set, and cluster all amplicons aligning to the same reference sequence. De novo OTU-generation uses pairwise clustering to assemble amplicons into groups—often with some sort of identity thresholding or difference metric. Subsampled (Sub) OTU generation [20] is a hybrid of the two techniques, starting with a closed strategy, and then taking all of the unmatched amplicons remaining and assembling them into OTUs via a de novo OTU-generation process.

To test OTU generation we took the amplicons generated from our 100 communities through QIIME, Mothur and a pplacer-based classification pipeline to generate OTUs. For QIIME, we attempted several different methods of OTU generation available in that package. Mothur uses a unique approach, including dereplication, alignment to the Silva reference database, further dereplication and finally clustering with Uclust; we consider this a closed strategy, given the discarding of sequences that do not align to the Silva reference. The pplacer-based pipeline uses an open OTU generation strategy via the swarm algorithm [21] (with pplacer itself agnostic to the OTU strategy and algorithm used).

For each amplicon we know the true origin organism. We can use this knowledge to ask if pairs of amplicons from the same organism are paired into OTUs by the classifier (true match), or not (false split). Similarly, for pairs of amplicons from different organisms we can ask if the pipeline correctly split these reads (true split), or incorrectly matched them into OTUs (false match). These results (with known true positives and true negatives, and tested outcomes for the same) are suited to the familiar sensitivity (true match over the sum of true match and false split) and specificity (true split over the sum of true split and false match) metrics used to evaluate tests. In this situation, sensitivity drops as incorrect splitting of amplicons increases. Conversely, specificity declines as amplicons are incorrectly matched by a pipeline.

**Fig. 2:**
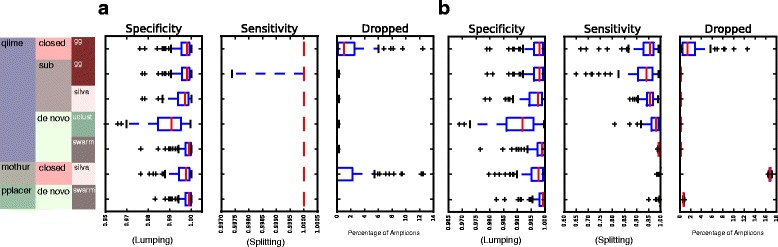
Assessment of OTU Performance. On the left are the various conditions tested. The first column specifies the pipeline, the second the strategy, the third the methodological details (e.g. reference set or algorithm used). Abbreviations: gg is GreenGenes. Sub is Subsetted OTU generation. **a** No sequencing error. **b** Simulated sequencing error

Figure 2 shows the distribution of sensitivity, specificity and percentage of amplicons dropped for the different pipelines, settings and strategies used for OTU generation for MiSeq data, without (Fig. 2a) and with simulated error (Fig. 2b), respectively, as a set of box-and-whiskers plots. Not surprisingly, in the idealized circumstance of perfect sequencing and PCR, the rate of false splitting of amplicons from the same organism into different OTUs was rare to non-existent, resulting in most sensitivities at 1. Specificity also approached 1, demonstrating that sequences from different organisms were only rarely lumped together. While the differences between settings and communities were significant by a paired Student’s T-test, the practical differences were slight.

With the addition of simulated sequencing errors in Fig. 2b, both the sensitivity (false splitting) and specificity (false matching) worsen, but remain modest. De-novo OTU generation with UCLUST-based methods consistently performed more poorly than Swarm-based methods, particularly as reflected by more incorrect splitting of amplicons during classification (statistically significantly different as compared to all other tested settings by a paired Student’s T-test with a target *p*-value of < 0.05).

With and without simulated sequencing error, closed OTU generation resulted in some dropped amplicons, a feature either non-existent or minimal in the sub or de novo OTU generation strategies.

### Classification

Classification is the process by which the clusters of amplicons generated in the OTU step are taxonomically assigned (and named). All of these pipelines take consensus amplicon sequences from each OTU, aligned against a set of (named) reference sequences; based on the alignment scores, names and taxonomies are selected for each OTU. Differences between pipelines arise in the selection of reference set, in how the alignments are completed and judged, and in how ties or similarly scoring alignments are settled with different names or taxonomies.

All of the source amplicons on our synthetic dataset have a name (almost exclusively to the species-level) and a defined taxonomy. For each true organism, we have a set of associated amplicons. Each of these amplicons can be: correctly classified (to the desired resolution, species or genus); under-called in the correct clade but not down to the desired rank; miscalled as a sibling, with the correct parent but wrong final identification (e.g. *Streptococcus intermedius* as *S. mitis*); overcalled down the right clade but overconfidently (e.g. as a strain when only a species should be called); miscalled down the entirely wrong clade; or dropped, and lost at this or an earlier stage.

Tables 1 and 2 summarize the performance of the pipelines using MiSeq data with simulated sequencing error, and targeting to species-level (Table 1) or genus-level (Table 2) resolution. Genus-level classification is correctly done for 50.4–74.8% of the source organisms, with QIIME, de-novo OTU generation and a curated subset of the Silva 123 reference set (as in Mothur) as the most successful strategy. Genus-level miscall rates varied from a low of 11.9–23.5%. Species-level classification was significantly less successful, (6.9–18.9% correct); when targeting species-level classification, miscall rates were comparable to those of genus-level targets (12.5–26.2%).

**Table 1:** Species Level Classification

Pipeline	OTU Strategy	OTU algorithm	Reference	Undercalled (%)	Undercalled (Ranks off)	Correct (%)	Misscalled (%)	Miscalled (Ranks off)	Lost (%)
QIIME	Closed		GreenGenes	55.8	1 (1–4)	18.9	22.3	4 (1–10)	3.0
QIIME	Sub	UClust	GreenGenes	63.3	1 (1–3)	12	24.5	4 (1–10)	0.2
QIIME	Sub	UClust	Silva	77.1	1 (1–6)	8.8	13.8	4 (1–14)	0.2
QIIME	De novo	UClust	GreenGenes	61.4	1 (1–3)	12.2	26.2	4 (1–10)	0.1
QIIME	De novo	UClust	Silva	77.7	1 (1–3)	8.7	13.4	4 (1–12)	0.1
QIIME	De novo	Swarm	GreenGenes	61.5	1 (1–4)	12.4	25.9	4 (1–10)	0.1
MOTHUR	Closed		Silva/RDP	54.6	1 (1–3)	6.9	21.9	10 (4–12)	16.6
pplacer	De novo	Swarm	RDP	68.2	1 (1–8)	18.1	12.5	4 (1–10)	1.2

Summary of Classification Performance. On the left are the various conditions tested. The first column specifies the pipeline, the second the OTU strategy, the third the methodological details (e.g. reference set or algorithm used). Table 1 is for species-level classification, Table 2 is for genus-level. Source organisms can be correctly called, undercalled (in the correct clade, but not the target species or genus level classification), or miscalled (placed down the wrong taxonomic clade). We present both the percentage in each category (correct, undercalled, and miscalled) and the median (min and max parenthetical) taxonomic ranks off for underacalled and miscalled source organisms

**Table 2:** Genus Level Classification

Pipeline	OTU Strategy	OTU algorithm	Reference	Undercalled (%)	Undercalled (Ranks off)	Correct (%)	Misscalled (%)	Miscalled (Ranks off)	Lost (%)
QIIME	Closed		GreenGenes	24.0	1 (1–3)	53.7	19.3	4 (1–9)	3.0
QIIME	Sub	UClust	GreenGenes	27.8	1 (1–3)	50.4	21.6	4 (1–9)	0.2
QIIME	Sub	UClust	Silva	11.6	1 (1–5)	74.8	13.4	4 (1–13)	0.2
QIIME	De novo	UClust	GreenGenes	22.9	1 (1–3)	53.5	23.5	4 (1–9)	0.1
QIIME	De novo	UClust	Silva	12.0	1 (1–3)	74.6	13.3	4 (1–11)	0.1
QIIME	De novo	Swarm	GreenGenes	26.3	1 (1–3)	50.5	23.1	5 (1–9)	0.1
MOTHUR	Closed		Silva/RDP	5	1 (1–2)	56.5	21.9	9 (1–11)	16.6
pplacer	De novo	Swarm	RDP	31.7	2 (1–7)	55.2	11.9	4 (1–9)	1.2

Summary of Classification Performance. On the left are the various conditions tested. The first column specifies the pipeline, the second the OTU strategy, the third the methodological details (e.g. reference set or algorithm used). Table 1 is for species-level classification, Table 2 is for genus-level. Source organisms can be correctly called, undercalled (in the correct clade, but not the target species or genus level classification), or miscalled (placed down the wrong taxonomic clade). We present both the percentage in each category (correct, undercalled, and miscalled) and the median (min and max parenthetical) taxonomic ranks off for underacalled and miscalled source organisms

Table 3 shows the relative performance of all the pipelines (and all data types) broken down by the order of the source organism. The ability of pipelines to correctly resolve organisms varied by the clade of the organism, particularly when considering the magnitude of error (by ranks off). Among the orders heavily represented in a typical stool sample, all pipelines struggled when attempting to classify *Enterobacteriales* and *Clostridiales*; performance for *Bacteroidales* was consistently stronger.

**Table 3:** Classification outcomes by order for all pipelines

Order	Percent				Ranks Off		
	Correct	Miscalled	Undercalled	Dropped	Miscalled	Undercalled	Total
Verrucomicrobiae	57.4	0.0	35.6	7.1	0.0	0.5	0.5
Lentisphaeria	30.9	0.0	57.5	11.5	0.0	1.3	1.3
Fusobacteriales	23.9	8.0	51.0	17.2	0.6	0.5	1.2
Acholeplasmatales	22.6	13.1	54.3	10.0	0.8	1.0	1.8
Pasteurellales	19.5	36.9	34.0	9.6	1.8	0.4	2.2
Bacteroidia	15.8	8.2	67.1	8.9	0.6	0.8	1.3
Lactobacillales	13.9	11.9	65.7	8.6	0.8	0.8	1.7
Selenomonadales	12.9	13.8	65.3	8.0	0.6	0.9	1.5
Mycoplasmatales	12.3	65.9	10.7	11.2	5.5	0.6	6.1
Clostridiales	10.0	30.9	48.7	10.5	1.8	0.8	2.6
Deltaproteobacteria	9.1	7.5	74.8	8.6	0.6	1.3	1.9
Burkholderiales	8.0	29.1	56.0	6.9	1.2	0.6	1.8
Actinobacteridae	7.8	15.2	70.1	6.9	1.0	0.8	1.8
Coriobacteridae	7.7	11.1	74.0	7.3	0.9	1.6	2.5
Erysipelotrichales	7.4	2.9	81.7	7.9	0.2	1.5	1.8
Enterobacteriales	2.2	37.9	50.0	10.0	2.3	1.0	3.3
Rhodospirillales	0.0	85.3	0.0	14.7	5.2	0.0	5.2

Classification Performance by Order of Source Organism. Combined performance for all pipelines and settings, broken down by the order of the organism. Correct are correctly classified organisms. Miscalled are organisms that are classified into the wrong clade. Undercalled are organisms placed into the correct clade, but at the higher order than species

Additional file 2: Figure S1 shows the true as compared to estimated relative abundance from three randomly selected synthetic communities and subjectively demonstrates the integrated effects of both misestimating in OTU generation and classification on complexity and composition of the community.

### Shannon index estimation

**Fig. 3:**
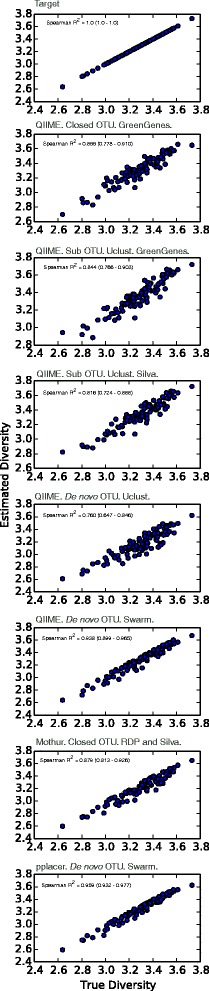
True versus Estimated Shannon Diversity. In each scatter plot, the x-axis is the true Shannon diversity for a community, and the y-axis is the estimated for the given pipeline. The top graph is true-versus-true for comparison in the others. We used Spearman’s correlations coefficients (inset, with 95% confidence intervals in *parentheses*) to test for monotonicity (consistency) of the estimates to true

The Shannon Index [22] is a commonly used metric for describing the alpha-diversity (both evenness and number of distinct organisms) of a community. Diversity is a key feature of microbial communities, and a meaningful way to compare communities. As diversity is mostly used as a comparator between communities, what we wish is for our estimates to be monotonic with the true diversity. To test how well each classifier estimates diversity, for each community we calculated a Spearman’s correlation coefficient when comparing the true diversity of the community to that estimated for a given pipeline as a test of monotonicity. Monotonicity is allows for systematic under or overestimation of true diversity, but retains the ability to accurately compare communities—and thus is a realistic and meaningful means of evaluating the pipeline output. Figure 3 graphically shows the results as scatter plots for MiSeq data with simulated error. The pplacer-based classifier achieved the best results with Spearman’s R^2^ of 0.96; the poorest performance was from Uclust-based de novo OTU generation, with a Spearman’s R^2^ of 0.77. Overall, de novo OTU generation via Swarm resulted in significantly better results (regardless of surrounding pipeline) than other methods (as determined by bootstrapped 95% confidence intervals from 1000 iterations with replacement).

### Pairwise distance estimation

The pairwise distance between two communities is a frequently used beta-diversity metric employed in clustering, multidimensional scaling, principle component analysis and other methods to demonstrate the relationships between communities. Again, as a comparator, ideally the estimated pairwise distance between communities would be monotonic as compared to the true pairwise distance. Some means of calculating distance consider the relationships between organisms phylogenetically when weighting the differences in their abundance, such as UniFrac [23] (weighted or not) and double principle coordinate analysis (DPCoA) [24]. The rationale is phylogenetically-related organisms contribute similar functions to communities and the functional similarity should be considered as part of a distance between communities. Weighted UniFrac has become the dominant method in the field for pairwise distance measurement.

**Fig. 4:**
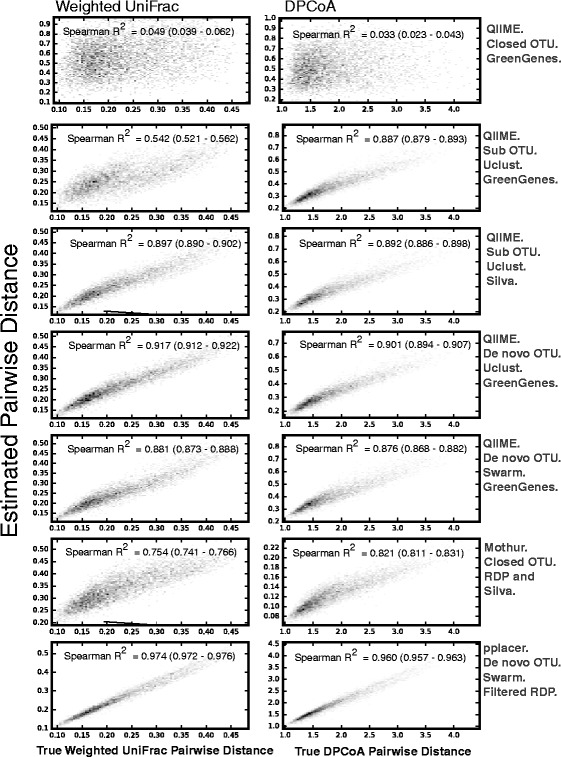
True versus Estimated Pairwise Distance. In each density plot, the x-axis is the true pairwise distance and the y-axis is the estimated pairwise distance between communities. We used Spearman’s correlations coefficients (inset, with 95% confidence intervals in *parentheses*) to test for monotonicity (consistency) of the estimates to true. The *left column* is pairwise distance as calculated by Weighted UniFrac distance. The *right column* is pairwise distances as calculated by double principle coordinate analysis (DPCoA)

We used the Spearman’s correlation coefficient to test the monotonicity between the true pairwise distance between communities and the estimated pairwise distance by the different pipelines. Figure 4 shows the results as a series of density plots for weighted UniFrac and DPCoA.

QIIME with closed OTU generation against the green genes database (the method described in the QIIME tutorial) has a distinctive method for phylogeny generation. As per the tutorial, one prunes the pre-made phylogenetic tree from greengenes (made from full length 16S sequences) down to the leaves recruited in the classification step. For the case of the pplacer-based pipeline, the recruited full-length 16S sequences are used to generate a de novo phylogeny. The other methods construct a de novo phylogeny from the amplicon sequences. The GreenGenes phylogeny performed distinctly and particularly poorly when compared to the true phylogenetic-based distance (based on the true full length 16S sequences from which the amplicons were generated assembled into a phylogeny with MG-RAST), regardless of distance metric (Spearman’s R^2^ of 0.049 or 0.033 for Weighted UniFrac or DPCoA respectively, as compared to all other settings resulting in a Spearman’s R^2^ of 0.54–0.97).

For settings resulting in a Spearman R^2^ around 0.7 (QIIME Sub OTU generation with the GreenGenes database for the closed portion, and Uclust for de novo and Mothur), DPCoA proved significantly more robust than weighted UniFrac. For setting resulting in a Spearman R^2^ in the 0.9’s (QIIME with de novo OTU generation by Uclust and the pplacer-based pipeline) weighted UniFrac was significantly better as a technique.

## Discussion

Amplicon-based approaches to describe complex microbial communities have theoretical limitations, including limited information available in some variable regions of taxonomically informative genes (like the 16S rRNA gene), and horizontal gene transfers scrambling the relationship between taxonomy and phylogeny. With a careful selection of a proper computational pipeline and settings for the pipeline one can achieve results close to theoretical limits for a given community type. A lack of close attention to these variables when selecting computational tools and settings can lead to skewed results.

Constructed communities remain an invaluable tool for optimizing methods for DNA storage, extraction, PCR and sequencing. DECARD and other in-silico techniques to generate a gold standard are complementary, with an ability to objectively evaluate the computational aspects of amplicon-based microbiome studies. In the current iteration, DECARD tests a relatively idealized circumstance in which there is no novel organism (organisms not represented in a reference set) in the communities. DECARD cannot assess how pipelines handle novel organisms, nor is it ideal for testing PCR or sequencing errors.

Even with these limits, for healthy human stool-like communities we discovered careful selection of reference sets, curation of reference sets and improved OTU generation techniques can all improve the accuracy of results. Shannon for alpha-diversity proved quite robust with the more optimal settings (e.g. Swarm-based de novo OTU generation). For beta-diversity, DPCoA was superior to weighted UniFrac when OTU generation was less robust.

Classification and taxonomic assignment to the species level remains a challenge for all of the pipelines, particularly in highly relevant orders like *Enterobacteriales* and *Clostridiales*. We hypothesize the clade-dependent performance to be primarily related to phylogenetic and taxonomic (or genomic) divergence in these clades—where the 16S sequence has less correlation with the overall function of the organism.

We were surprised at the significant challenges in classification. In our preliminary studies, we used 16S SSU rRNA exclusively from reference organisms or complete genomes to generate our synthetic reads without simulated PCR or sequencing errors; even in this very idealized circumstance, classification success was limited in a similar way to the data presented here.

We speculate duplicated, misannotated and imperfectly sequenced entries in reference databases contribute to classification errors. Further, an amplicon sequence can match multiple reference database entries with different taxonomic classifications, due to duplicated sequences and the amplicon region sequence being shared between distinct full-length sequences. How a pipeline handles this ambiguity can affect the result quality. We favor classifiers that reflect the ambiguity and offer higher rank classifications in this situation.

It’s imperative for reproducibility and interpretability of results that researchers include the specific method details in microbiome studies: the version of the software used; the specific OTU-generation strategy (closed, de novo, sub, etc.) and details (algorithm and reference database, including version or date); and the specific tactic used for classification and the version or date of the reference set selected. We demonstrate here that seemingly minor differences in these details can have a meaningful and statistically significant impact on the validity of the outputs. It is insufficient for good science to simply specify the software pipeline used. Nor is it sufficient to use the settings in the tutorials or standard operating procedures of a computational pipeline and assume the results will be optimal.

We demonstrate here that with some optimization of the settings selected, the amplicon-sequence based estimation of microbial communities remains a valuable technique. But investigators should strive to optimize the reliability of their results and understand how the computational pipeline selected and specific settings chosen may influence results as they design and interpret experiments.

## Conclusion

Amplicon-based methods for describing complex microbial communities can be accurate and precise, but only with careful attention to settings and method details. Synthetic datasets and constructed communities will help researchers select these settings and details. The methods and classification details must be included when microbiome studies are published to ensure reproducibility and validity.

## Methods

### Reference sequence curation

Near-full length (>1000 bp) 16S ribosomal rRNA sequences with no ambiguous bases were acquired from the NCBI 16S microbial (downloaded on April 21 2016) and Silva 16S (version 123) rRNA databases. Sequences were categorized to genus and then species. When multiple sequences were available for a given species, all of the sequences for a given species were clustered and outliers dropped—defined as sequences greater than the 90th percentile in distance from the nearest centroid using the deenurp [25] package in filter outlier mode.

### Stool microbiome estimation

The mean and standard deviation of relative abundance of genera from a random selection 100 of stool microbiomes from the NIH Human Microbiome Project and from healthy hematopoietic stem cell donors were used to determine the composition of a typical stool microbiome.

### Defined community creation

The generate_targets.py module picks specific sequences and their relative abundance to generate communities. A CSV file is taken as an input to define the community characteristics; each row is a genus, with a targeted mean and standard deviation for fractional abundance. Each genus is also given parameters, either a mean and standard deviation number of species to be included for this genus, or parameters (a, b) for the log function:

$$ n= a* \log (f)+ b $$

Where *n* is the number of species, *f* is the fractional abundance of this organism in the community.

Using these parameters, the module selects specific reference sequences, and then calculates the fraction of the community that this specific reference sequence (and organism) represents.

### In-silico PCR and amplicon generation

The generate_sequences.py module of DECARD takes the target file generated in the community creation step, a desired read depth and a FASTA file containing the primer sequences and performs in-silico PCR to generate amplicons with a known origin. For simulated 454 sequencing, we used a read depth of 5000 reads per community, and the human microbiome project (HMP) primers (F (357F): CCTACGGGAGGCAGCAG. R (926R): CCGTCAATTCMTTTRAGT). For Illumina MiSeq simulations, we used a read depth of 50,000 per community, and the EMP primers (F (U515F): GTGYCAGCMGCCGCGGTAA. R (806R): GGACTACNVGGGTWTCTAAT).

For each reference read in the target file, the number of reads is calculated by multiplying the target fractional abundance by the read depth. Provided the rounded value is at least one, in-silico PCR is performed by aligning primer sequences to the reference sequence, testing for annealing at the 3′ end of the primer and a sufficient degree of sequence similarity. Amplicons are then taken by slicing from the 5′ to 3′ primer, a unique ID is generated, and the combination stored in FASTA format in a new file. Separately, a mapping file is generated connecting the sequence ID to a source reference accession, organism and taxonomy.

### Error generation

The resultant amplicon files are run through the ART [26] to simulate sequencing errors. art_454 was used for 454-style sequencing. We used our own recent 454 data to build a new error model (available in supplemental materials). For Illumina MiSeq style data, art_illumina was used to generate simulated paired-end reads with a length of 250 bp, using the built-in MiSeq error model. For each simulated amplicon, one read with sequencing error was generated.

### Calculation of species diversity of real and synthetic data

As per [19], we used the formula:

$$ q D = {\left({\displaystyle \sum_{i=1}^S}{p}_i^q\right)}^{\raisebox{1ex}{$1$}\!\left/ \!\raisebox{-1ex}{$1- q$}\right.} $$

Where qD is the species diversity, q is the Hill number, S is the number of organisms, *p*_*i*_ is the relative abundance of organism *i*. For q = 1, we took the limit of q = 1. To calculate 95% confidence intervals, we bootstrapped with replacement 5000 iterations.

### QIIME classification

Quantitative Insights Into Microbial Ecology (QIIME) [1] open-source software (version 1.9.1) was used following the standard operating procedures on the website. The default QIIME settings for preprocessing were used, including filtering out sequences that had any ambiguous bases or homopolymer runs longer than 6. For simulated 454 sequences, the length requirement was modified to be between 200 and 1000 and a more lenient maximum ambiguous base of 6. The communities where errors were introduced had either a minimum average quality score of 25 or a minimum Phred quality score of three and truncation at three consecutive poor quality base calls.

We used three OTU picking strategies with default parameters: de novo, closed and subsampled open-reference [20]. In de novo, sequences are clustered into centroids with each cluster fulfilling the 97% identity with Uclust version 1.2.22q [27] or with a local difference of one with Swarm [21]; a representative sequence for each OTU is aligned with PyNAST [28] to a reference set for taxonomy assignment, either GreenGenes [29] version 13.8 or Silva version 119 [30]. In closed OTU picking, sequences were queried against the reference database (Greengenes version 13.8) at the default 97% identity with Uclust for clustering, Uclust classifier with Silva version 119 (97% OTU), or Swarm classifier with Greengenes (version 13.8). In sub-sampled open-reference OTU picking, sequences were first queried against the reference database (Greengenes version 13.8) and if matched they were classified with Uclust (fast uclust settings). From the pool of sequences that did not match a reference OTU at greater than 97% percent identity, 0.001% sequences were subsampled and clustered de novo. These cluster centroids were used as new reference OTUs for the remaining pool of sequences that had not matched an OTU in the reference database. Alternative runs of subsampled open reference OTU picking included using Silva version 119 as reference database.

### MOTHUR classification

Mothur [2] (version 1.36.1) was employed following the standard operating procedures from the website. For preprocessing the sequences were screened for having no ambiguous bases and maximum homoploymer run 8. In the communities with simulated error we combined the paired end reads with all quality scores higher than 25 considered acceptable, and used a 50-bp sliding window (miseq data) or trim sequence with average quality score drops below 30 over a 50 base window (454 data). The preprocessed sequences were de-duplicated and aligned to a 50,000–column wide SILVA-based reference database (Silva version 123, previously trimmed to the section of 16S rRNA genes amplified by the PCR primer used to generate the amplicons) using a NAST-based aligner.

Aligned sequences were filtered to remove any sequences that contain just gaps, and this was done prior to deduplication and a merge of all sequences that had two or fewer base pairs different. Next chimeras (which were defined as having at least three bases more similar to a chimera of reference sequences than to a single reference sequence) were identified with Uchime [31] and removed. Finally sequences were classified with RDP [32] version 14 with a bayesian classifier (RDP) with a kmer size of 8, 100 iterations and a cutoff of 80% bootstrap value for taxonomic assignment.

### pplacer classification

This classification was done as in [33], using a pplacer-based pipeline. The 14.0 revision of the RDP reference database [32] (in turn culled from the NCBI databases) was broken down into reference sequences with well-formed species names (e.g. genus, species) and those without names (e.g. ‘uncultured bacterium’) using the deenurp package. Potentially mis-annotated reference sequences were identified using “deenurp filter_outliers” using the default parameters on the basis of within-species pairwise distances and discarded. Only the named references were used for the subsequent steps.

The synthetic reads were first clustered into OTUs via simple dereplication for 454 reads (combining identical sequences) or with Swarm [21] for MiSeq reads to a local difference of 1, and dropping of singleton clusters. The resultant representative sequences were then used to recruit sequences from the named reference set (using “deenurp select_references” with default parameters). Following recruitment of reference sequences, species that were the only representatives of a genus were identified as “lonely” taxa; additional reference sequences representing closely related species from the same genus were added (using “deenurp fill_lonely” with default parameters) to provide additional taxonomic context. pplacer [18] was then used to place the representative sequence reads onto the reference tree. The placed sequences were then classified using guppy (part of the pplacer package) with the ‘hybrid2’ classifier.

### Standardization to a common output format

For each classification pipeline considered, DECARD has modules that convert the pipeline output to a common table mapping each sequence to an OTU and classification. This OTU table is in CSV format with the following headers:

seq,community,otu_id,ncbi_rank,name,ncbi_tax_id,taxonomy_string,weight

### Testing of OTU generation

The OTU tables can be compared to the mapping file with the test_otu.py module of DECARD. For each pair of sequences, we determined if they were or were not from the same source, and then determined if the classifier appropriately split or matched the sequences in the OTU generation step. For each community, we used the results of these pairwise tests to determine specificity (truly from different sources divided by the sum of truly from different sources + incorrectly matched pairs) and sensitivity (truly from the same source divided by the sum of truly from the same source and incorrectly split pairs) of the OTU generation step.

### Assessment of classification accuracy and precision

The test_classification.py module of DECARD takes the OTU output, the mapping file and a target rank (species or genus) and then scores the classification performance. Sequences are grouped by their source accession. The classification of the sequences is compared to their true source, and scored as visually described in Additional file 3: Figure S2.

### Shannon diversity and pairwise distance calculation

Shannon diversity and pairwise distance calculations were completed via the Phyloseq package in R. [34]. For tree-based distance metrics (UniFrac, DPCoA), a phylogeny was generated to be used as a ‘true’ phylogeny with RaXML from an alignment generated by cmalign of the full length source 16S SSU rRNA sequences from which the amplicons were generated in-silico to create the community.

## Additional files

Additional file 1: Table S1–S4. Community composition definitions. (ZIP 11 kb)

Additional file 2: Figure S1. True versus Estimated Relative Abundance for Three Synthetic Communities. Each band represents one organism. The height of each band of color is proportional to the relative abundance of sequence types. Taxonomically similar organisms are closer in color. Colors are by phylum (inspired by a gram stain): Blue and purple for *Firmucutes*; orange for *Bacteroides*; Tan and pinks for *Proteobacteria*. The left-most column for each community is the true composition of the community. The second column is as estimated by Mothur, the second as by our internal pplacer-based classifier, the third as by QIIME with Uclust-based de novo OTU generation and then classification against Silva, the fourth QIIME with closed OTU generation and classification against the GreenGenes reference. (EPS 615 kb)

Additional file 3: Figure S2. Possible Classification Outcomes. (EPS 1039 kb)

## Abbreviations

GG: GreenGenes
HMP: Human Microbiome Project
OTU: Of operational taxonomic unit
PCR: Polymerase chain reaction
QIIME: Quantitative Insights Into Microbial Ecology
Sub: Subsetted OTU generation

## References

[1] Caporaso JG, Kuczynski J, Stombaugh J, Bittinger K, Bushman FD, Costello EK (2010). QIIME allows analysis of high-throughput community sequencing data. Nat Methods.

[2] Schloss PD, Westcott SL, Ryabin T, Hall JR, Hartmann M, Hollister EB (2009). Introducing mothur: open-source, platform-independent, community-supported software for describing and comparing microbial communities. Appl Environ Microbiol.

[3] Brooks JP, Edwards DJ, Harwich MD, Rivera MC, Fettweis JM, Serrano MG (2015). The truth about metagenomics: quantifying and counteracting bias in 16S rRNA studies. BMC Microbiol.

[4] Lee CK, Herbold CW, Polson SW, Wommack KE, Williamson SJ, McDonald IR (2012). Groundtruthing next-gen sequencing for microbial ecology-biases and errors in community structure estimates from PCR amplicon pyrosequencing. PLoS One.

[5] Schloss PD, Gevers D, Westcott SL (2011). Reducing the effects of PCR amplification and sequencing artifacts on 16S rRNA-based studies. PLoS One.

[6] D’Amore R, Ijaz UZ, Schirmer M, Kenny JG, Gregory R, Darby AC (2016). A comprehensive benchmarking study of protocols and sequencing platforms for 16S rRNA community profiling. BMC Genomics.

[7] Gohl DM, Vangay P, Garbe J, MacLean A, Hauge A, Becker A (2016). Systematic improvement of amplicon marker gene methods for increased accuracy in microbiome studies. Nat Biotechnol.

[8] Thorsen J, Brejnrod A, Mortensen M, Rasmussen MA, Stokholm J, Al-Soud WA (2016). Large-scale benchmarking reveals false discoveries and count transformation sensitivity in 16S rRNA gene amplicon data analysis methods used in microbiome studies. Microbiome.

[9] Bokulich NA, Rideout JR, Mercurio WG, Shiffer A, Wolfe B, Maurice CF (2016). mockrobiota: a Public Resource for Microbiome Bioinformatics Benchmarking. mSystems.

[10] White JR, Navlakha S, Nagarajan N, Ghodsi M-R, Kingsford C, Pop M (2010). Alignment and clustering of phylogenetic markers—implications for microbial diversity studies. BMC Bioinformatics.

[11] Schloss PD (2010). The effects of alignment quality, distance calculation method, sequence filtering, and region on the analysis of 16S rRNA gene-based studies. PLoS Comput Biol.

[12] Schloss PD, Westcott SL (2011). Assessing and improving methods used in operational taxonomic unit-based approaches for 16S rRNA gene sequence analysis. Appl Environ Microbiol.

[13] Schmidt TSB, Matias Rodrigues JF, von Mering C (2015). Limits to robustness and reproducibility in the demarcation of operational taxonomic units. Environ Microbiol.

[14] Wang X, Cai Y, Sun Y, Knight R, Mai V (2012). Secondary structure information does not improve OTU assignment for partial 16 s rRNA sequences. ISME J.

[15] Klindworth A, Pruesse E, Schweer T, Peplies J, Quast C, Horn M (2013). Evaluation of general 16S ribosomal RNA gene PCR primers for classical and next-generation sequencing-based diversity studies. Nucleic Acids Res.

[16] Hong S, Bunge J, Leslin C, Jeon S, Epstein SS (2009). Polymerase chain reaction primers miss half of rRNA microbial diversity. ISME J.

[17] Kozlov AM, Zhang J, Yilmaz P, Glöckner FO, Stamatakis A (2016). Phylogeny-aware identification and correction of taxonomically mislabeled sequences. Nucleic Acids Res.

[18] Matsen FA, Kodner RB, Armbrust EV (2010). pplacer: linear time maximum-likelihood and Bayesian phylogenetic placement of sequences onto a fixed reference tree. BMC Bioinformatics.

[19] Chao A, Chiu C-H, Jost L (2014). Unifying Species Diversity, Phylogenetic Diversity, Functional Diversity, and Related Similarity and Differentiation Measures Through Hill Numbers. Annu Rev Ecol Evol Syst.

[20] Rideout JR, He Y, Navas-Molina JA, Walters WA, Ursell LK, Gibbons SM (2014). Subsampled open-reference clustering creates consistent, comprehensive OTU definitions and scales to billions of sequences. PeerJ.

[21] Mahé F, Rognes T, Quince C, de Vargas C, Dunthorn M (2014). Swarm: robust and fast clustering method for amplicon-based studies. PeerJ.

[22] Shannon CE (1948). A Mathematical Theory of Communication. Bell Syst Tech J.

[23] Lozupone C, Knight R (2005). UniFrac: a new phylogenetic method for comparing microbial communities. Appl Environ Microbiol.

[24] Pavoine S, Dufour A-BA-B, Chessel D (2004). From dissimilarities among species to dissimilarities among communities: a double principal coordinate analysis. J Theor Biol.

[25] McCoy C, Hoffman N, Rosenthal C, Matsen F. deenurp: 16S rRNA gene sequence curation and phylogenetic reference set creation. [Internet]. 2014. Available from: https://github.com/fhcrc/deenurp. Accessed 15 Mar 2017.

[26] Huang W, Li L, Myers JR, Marth GT (2012). ART: a next-generation sequencing read simulator. Bioinformatics.

[27] Edgar RC (2010). Search and clustering orders of magnitude faster than BLAST. Bioinformatics.

[28] Caporaso JG, Bittinger K, Bushman FD, DeSantis TZ, Andersen GL, Knight R (2010). PyNAST: a flexible tool for aligning sequences to a template alignment. Bioinformatics.

[29] DeSantis TZ, Hugenholtz P, Larsen N, Rojas M, Brodie EL, Keller K (2006). Greengenes, a chimera-checked 16S rRNA gene database and workbench compatible with ARB. Appl Environ Microbiol.

[30] Quast C, Pruesse E, Yilmaz P, Gerken J, Schweer T, Yarza P (2013). The SILVA ribosomal RNA gene database project: improved data processing and web-based tools. Nucleic Acids Res.

[31] Edgar RC, Haas BJ, Clemente JC, Quince C, Knight R (2011). UCHIME improves sensitivity and speed of chimera detection. Bioinformatics.

[32] Maidak BL, Olsen GJ, Larsen N, Overbeek R, McCaughey MJ, Woese CR (1997). The RDP (Ribosomal Database Project). Nucleic Acids Res.

[33] Srinivasan S, Hoffman NG, Morgan MT, Matsen FA, Fiedler TL, Hall RW (2012). Bacterial communities in women with bacterial vaginosis: high resolution phylogenetic analyses reveal relationships of microbiota to clinical criteria. PLoS One.

[34] McMurdie PJ, Holmes S (2013). phyloseq: an R package for reproducible interactive analysis and graphics of microbiome census data. PLoS One.

